# Opportunities and Challenges*:* Hepatitis C Testing and Treatment Access Experiences Among People in Methadone and Buprenorphine Treatment During COVID-19, Arizona, 2021

**DOI:** 10.1016/j.focus.2022.100047

**Published:** 2022-11-05

**Authors:** Beth E. Meyerson, Danielle M. Russell, Missy Downer, Amirah Alfar, Irene Garnett, John Lowther, Rebecca Lutz, Arlene Mahoney, Julie Moore, Greg Nuñez, Savannah Samorano, Benjamin R. Brady, Keith G. Bentele, Brenda Granillo, Arizona Drug Policy Research & Advocacy Board

**Affiliations:** 1 Family & Community Medicine, College of Medicine Tucson, The University of Arizona, Tucson, Arizona; 2 Southwest Institute for Research on Women, College of Social & Behavioral Sciences, The University of Arizona, Tucson, Arizona; 3 School of Social Transformation, Arizona State University, Tempe, Arizona; 4 Southwest Recovery Alliance, Phoenix, Arizona; 5 Drug Policy Research & Advocacy Board, The University of Arizona, Tucson, Arizona; 6 CAN Community Health Services, Phoenix, Arizona; 7 Comprehensive Pain and Addiction Center, The University of Arizona, Tucson, Arizona; 8 Mel & Enid Zuckerman College of Public Health, The University of Arizona, Tucson; Arizona

**Keywords:** Hepatitis C, HCV screening, HCV curative treatment, MOUD provider health services, HCV cascade

## Abstract

**Introduction:**

The purpose of this study was to characterize hepatitis C virus screening and treatment access experiences among people in treatment for opioid use disorder in Arizona during COVID-19.

**Methods:**

Arizonans receiving treatment for opioid use disorder from methadone clinics and buprenorphine providers during COVID-19 were interviewed about hepatitis C virus testing, curative treatment, and knowledge about screening recommendations. Interviews were conducted with 121 people from August 4, 2021 to October 10, 2021. Qualitative data were coded using the categories of hepatitis C virus testing, knowledge of screening recommendations, diagnosis, and experiences seeking curative treatment. Data were also quantitated for bivariate testing with outcome variables of last hepatitis C virus test, diagnosis, and curative treatment process. Findings were arrayed along an adapted hepatitis C virus cascade framework to inform program and policy improvements.

**Results:**

Just over half of the sample reported ever having tested for hepatitis C virus (51.2%, *n*=62) and of this group, 58.1% were tested in the past 12 months. Among those who were ever tested, 54.8% reported a hepatitis C virus diagnosis and 16.1% reported either being in treatment or having been declared cured of the hepatitis C virus. Among those who were diagnosed with hepatitis C, 14.7% indicated that they unsuccessfully tried to access curative treatment and would not attempt to again. Reasons cited for not accessing or receiving curative treatment included beliefs about treatment safety, barriers created by access requirements, natural resolution of the infection, and issues with healthcare coverage and authorization.

**Conclusions:**

Structural barriers continue to prevent curative hepatitis C virus treatment access. Given that methadone and buprenorphine treatment providers serve patients who are largely undiagnosed or treated for hepatitis C virus, opportunities exist for them to screen their patients regularly and provide support for and/or navigation to hepatitis C virus curative treatment.

## HIGHLIGHTS

• Medication for opioid use disorder (MOUD) providers can screen patients often and navigate to hepatitis C virus (HCV) curative treatment.
• For screening value, 55% of MOUD patients were diagnosed with HCV in the past 2 years.
• Integration of HCV services into a MOUD environment requires a culture shift.
• Patient-centered/empowered models can assist HCV integration in MOUD treatment.
• HCV cure can be assured with treatment navigation or provision by MOUD clinics.

## INTRODUCTION

Since 2014, the possibility of hepatitis C virus (HCV) elimination has emerged because of safe and effective oral curative treatment.^1^ Community-based rapid screening among populations at risk for HCV helped to characterize the need for innovative public health approaches and policy changes through reported implementation challenges and successful screening, which more accurately defined the need for HCV curative treatment. Although some U.S. states strengthened systems and policies to eliminate HCV in their communities,^2^ differences in state health policy environments continue to hamper national efforts to eliminate HCV. Barriers to HCV treatment in the U.S. include state Medicaid and insurance policies requiring, for example, preauthorization and abstinence from all illicit drug use^3^; high drug prices that challenge state budgets^4^; requirements for specialist referral; limitations based on liver disease severity^5^; and sociostructural stigma against people who use drugs.^6^ These challenges have likely contributed to ongoing HCV outbreaks in at least 30 states.^7^ Opportunities for HCV elimination depend on our ability to identify and address these issues within the states themselves.

Arizona is among the states striving to eliminate HCV. For example, Arizona hired additional HCV staff and initiated a statewide elimination planning effort that is both transdisciplinary and community-based. Although efforts there have been initially lauded nationally by the National Viral Hepatitis Roundtable in 2021, more improvement is needed, as Arizona received a failing grade owing to access barriers to curative treatment in 2022. This includes collecting data to characterize screening and treatment needs, policy changes to remove structural barriers, and more robust evaluation of public health interventions to achieve HCV elimination. In clinical settings, providers continue to impose liver damage requirements and sobriety restrictions (despite state discouragement of this practice) and require prescriptions for curative treatment by specialists.^8^ It is possible that provider behavior is structurally defined by payer decisions. The degree to which this is the case needs to be clarified in Arizona.

Nationally, studies focused on HCV screening have consistently found that general population screening is cost effective but insufficient because it does not fully identify the populations who need curative treatment, especially when interventions to improve treatment access are not in place.^9^^,^^10^ It is also often overlooked that to be cost effective, general population‒based screening must reach subpopulations that do not ordinarily seek health care. This includes people who use drugs, who are regularly underserved and even mistreated by healthcare providers.^11^ Community-based screening programs by local organizations serving people who use drugs have been found to be an effective means of identifying greater numbers of people who need treatment.^12^ In Arizona, a recent statewide study of a community-based harm reduction organization HCV screening program among people who use drugs identified an HCV reactive rate of 21%. This rate far exceeded that found by other methods of HCV screening in Arizona.^13^^,^^14^

It may be possible to improve the levels of HCV identification and curative treatment by involving opioid use disorder (OUD) providers in HCV screening and linkage to or provision of curative treatment. However, this approach would be limited by current regulatory barriers and the fact that the culture of OUD treatment―especially methadone―is unlike health care and more like carceral programs rife with surveillance and without a patient-centered focus.^15^^,^^16^ Methadone treatment is particularly organized this way in the U.S. because patients are supervised in dosing, are supervised in urine testing, and have little voice relative to their ability to adjust treatment frequency or dosing without the risk of losing the treatment that is termed (in this setting) as privileges.^17^ There are few innovative models. One New South Wales study found that integrating HCV curative treatment in methadone clinics appeared to transform the framework of care―moving it away from the carceral frame to more of a healthcare delivery frame.^18^ Because HCV antibody prevalence is high among people who are methadone patients (67%–96%),^19^ integrating curative services in OUD treatment settings may advance national efforts to eliminate HCV.

In Arizona and elsewhere,^20^^,^^21^ people who use drugs often face discrimination when trying to access primary health care generally.^10^ This discrimination provides impetus to understand whether OUD treatment settings are appropriate places to systematize HCV screening and curative treatment provision or navigation. To explore this potential, we first sought to describe the HCV testing and curative treatment experiences of those on medication for OUD (MOUD) during the coronavirus disease 2019 (COVID-19) period in Arizona.

## METHODS

### Study Sample

We conducted interviews with 121 people living in Arizona who were aged ≥18 years and receiving buprenorphine or methadone treatment for OUD during COVID-19 (January 2020–March 2021). The study was conducted between August 4, 2021 and October 10, 2021 in communities throughout Arizona.

A total of 18 people with lived experience of drug use were recruited by the Arizona-based Drug Policy Research & Advocacy Board and harm-reduction organizations to recruit for and conduct interviews with MOUD patients. These field interviewers were trained, paid, and supported to conduct 60-minute, face-to-face, audio-recorded interviews with a socially recruited convenience sample of people who received OUD treatment in Arizona at some point during the COVID-19 period. Interviewers were hired throughout the state, from urban and rural areas, and with bilingual Spanish/English skills.

### Measures

The findings reported in this study were part of a larger study of patient experiences with methadone and buprenorphine access during COVID-19 period.^17^ Because the parent study focused on OUD treatment access to methadone and buprenorphine during COVID-19 period, we measured where people received their medication during this time period by type of clinic (methadone clinic or buprenorphine provider). The interview instrument contained 27 questions that were both qualitative and quantitative. Because this was a community-based participatory and action study, the Arizona-based Drug Policy Research & Advocacy Board developed the interview instrument, which was then considered and edited by the interviewer cohort. These steps assured that the study would gather information that was both important and meaningful to the community of people who use(d) drugs. This was a mixed-methods study with a primarily qualitative emphasis on exploring the policy experience of the interview cohort. Quantitative elements measuring discrete experiences and characteristics of treatment were included, and quantitation of qualitative themes was anticipated. Instrument items measured demographics, treatment experiences, treatment providers, services offered during the COVID-19 period, health risks for COVID-19, health issues related to drug use, healthcare experience, last HCV test, knowledge about HCV screening recommendations for people who use drugs, and HCV curative treatment experiences. The current screening recommendations in settings where HCV prevalence is >0.1% include (1) screening at least once in a lifetime for all adults aged ≥18 years, (2) screening for all pregnant persons during each pregnancy, and (3) periodic testing for all persons with risk factors regardless of risk disclosure. This includes persons who inject drugs and share syringes or drug preparation equipment.^22^ Interviews were conducted in Spanish and English and were transcribed by the principal investigator (BEM) to text for analysis. The period of COVID-19 was defined as being between January 2020 and March 2021.

### Statistical Analysis

Interview data were coded using an a priori coding framework including HCV testing, HCV testing location, HCV treatment experience, and reported HCV diagnoses in the past 2 years. Emerging themes describing barriers to treatment were also coded. Data were selected for quantification to allow frequency and distribution reporting and for tests of association where possible with bivariate (yes/no) outcome variables, including last HCV test, HCV diagnosis (yes), and in or completed HCV curative treatment if diagnosed. Data were coded into 3 categories: within the last 6 months, within the last 7–12 months, and >12 months before the interview. Data were then arranged using an adapted hepatitis C cascade, including testing through curative treatment.^11^ The HCV treatment cascade is a useful framework for this purpose and allows for the visualization of important components in the path toward HCV elimination. Human subjects oversight was provided by the University of Arizona IRB, and overall study direction was provided by the Drug Policy Research & Advocacy Board, a transdisciplinary statewide group comprised OUD providers, people with lived experience (in or not in OUD treatment), harm-reduction providers, and university-based researchers.

## RESULTS

The study included 121 people from 11 Arizona cities and towns, representing urban (36.4%), rural (17.4%), and mixed urban/rural (46.3%) communities. Participant ages ranged from 19 to 67 years, with a mean age of 38.1 years (SD=11.1 years). The sample was predominantly non-Hispanic White (66.1%). About one quarter of participants (24.0%) were of Hispanic (White/Black/multiracial) ethnicity, and 3.3% were respectively from indigenous (Pascua Yaqui, Navajo, Apache, Chiricahua) communities, Black communities, and Asian (Korean, Japanese, not identified) communities. The sample was primarily cisgender, with cisgender males comprising 61.1% of the sample, cisgender females comprising 36.4%, and nonbinary participants comprising 2.5%. The vast majority of the sample (83.5%) identified as heterosexual, and 10.7% identified as bisexual. Lesbian, gay, pansexual, and queer participants comprised 5.8% of the sample.

Over half of the sample (67.8%, *n*=82) received treatment from a methadone clinic, 20.7% (*n*=25) received buprenorphine from either a clinic or clinician in private practice, and 11.6% (*n*=14) received both methadone and buprenorphine from different providers during the period of study. Methadone in the U.S. usually requires daily supervised dosing, although during the COVID-19 period, the U.S. federal government allowed flexibility, so some providers would permit multiday take-home dosing. Buprenorphine is dosed by prescription on a near-monthly basis.^23^

### Hepatitis C Virus Testing

“I don't know where to go (to get tested). Usually (I get tested) when I leave jail or go to jail…..I saw the [MOUD] clinics, they do it when you first start” (Participant 101, Phoenix, AZ).

Just over half of the sample (51.2%, *n*=62) reported ever testing for HCV. Participants indicated the time of their most recent test. Table 1 shows the last reported HCV test among interview participants.

**Table 1:** Most Recent HCV Test Among People in Methadone or Buprenorphine Treatment Reporting Having Had an HCV Test, Arizona, 2021 (*n*=62)

Time frame	*n* (%)
Tested in the last 6 months	24 (38.7)
Tested in the last 7–12 months	12 (19.4)
Tested >12 months ago	26 (41.9)

HCV, hepatitis C virus.

There were no statistically significant associations between the most recent test and OUD provider type. However, among the 59 people who reported never testing for HCV, over half (58.3%) were in methadone treatment (chi-square=0.28, *p≤*0.05).

### Hepatitis C Virus Screening Knowledge

Participants reported having limited knowledge about HCV screening recommendations for people who use drugs. Less than 10% (9.9%, *n*=12) knew that people who use drugs or those who inject them should be screened every 3 months, 6 months, or regularly. All 3 answers were accepted as accurate given the mixture of messages about what regularly might mean. Another 29.9% (*n*=35) stated that they did not know the recommendations for HCV screening: “You know, I honestly don't know. Back when we started on methadone, we were using heroin intravenously… but I would assume that there were some [testing] requirements back then. And I don't know what they were because I just wasn't in that particular circle” (Participant 94, Tucson, AZ).

Participants reported receiving their last HCV test at a variety of locations. For those screening >12 months before the interview, their last HCV test was administered at methadone program entry, at entry or exit from jail or prison, during hospitalization, or at a plasma center. Those who reported testing more recently tended to report testing at their methadone clinic or a local harm-reduction outreach program (Table 2). “I think that the needle exchange…. did it, but I'm not sure….. I don't know the doctors….Most people don't go to the doctors that are using” (Participant 40, Kingman, AZ).

**Table 2:** Location of the Last Hepatitis C Test Among People on Methadone or Buprenorphine During COVID-19, Arizona, 2021 (*n*=121)

Location	Number reporting	% total sample	% total reporting HCV testing, *n*=62
MOUD clinic (methadone and/or buprenorphine)	19	15.7	30.6
Hospital or urgent care	13	10.7	21.0
Primary care provider or clinic	10	8.3	16.1
Jail or prison	7	5.8	11.3
Syringe service program	6	5.0	9.7
Detox program	4	3.3	6.5
Did not recall	3	2.5	4.8
Did not report testing for HCV	59	48.7	–

HCV, hepatitis C virus; MOUD, medication for opioid use disorder.

Among all participants, 36 (29.8%) reported being diagnosed with HCV in the past 2 years. This represents a 58.1% positivity rate among those reporting an HCV test. Of those reporting an HCV test, fewer than 5 people reported already knowing that they were HCV positive at the time they tested: “Yeah, I was screened one month or two months ago, but I've known [that I've had HCV] for several times now.” (Participant 116, Somerton, AZ)

### Hepatitis C Virus Treatment Experiences

Among the sample, 10 people reported either being in treatment or having been declared cured of HCV. This represented 8.3% of the sample and 29.4% of those diagnosed with HCV. When the 36 people diagnosed with HCV were asked about whether they sought HCV curative treatment, 10 indicated that they were not seeking treatment, and 3 indicated that they were still trying to access curative treatment. No one was able to access HCV curative treatment at their testing location, whether MOUD provider or otherwise. Thus, treatment was referred out from the screening provider in every case. Table 3 shows responses and exemplar statements by interview participants that represent the reasons for accessing or not accessing treatment.

**Table 3:** HCV Treatment Access Among People on Methadone or Buprenorphine During COVID-19 Who Were Diagnosed With HCV in the Past 2 Years, Arizona 2021 (*n*=34)

Response	*n* (%)	Exemplar statement
Not accessing treatment		
Does not want treatment	3 (8.8)	I've heard some stories that it can make you sick. So I just don't, I'm a big chicken. (Participant 130, Prescott) I have numerous friends that I have watched that … get the hepatitis C cure. It cured them with Hepatitis, but they turned around and ended up with…. liver cancer and one of them got kidney cancer. (Participant 38, mesa)
Cannot get it and not trying again	5 (14.7)	Participant: I tried to get it, but I was dirty [positive UA]. (Participant 122, Yuma) Interviewer: Yeah… I actually got denied because my liver wasn't damaged enough.…. Can you believe that?
Doesn't need it anymore	2 (5.9)	This last time when I was in prison, the doctor told me that my hepatitis C was pretty much gone. There was no kidney damage, no liver damage…. But they could still find it on a mass spec, but it wasn't affecting me because I wasn't using, and it's a rare genotype that people get that basically it cures itself, but it's not gone. It just doesn't affect you like it would if you were there using and your kidneys were failing and stuff like that. (Participant 7, Tucson) It wasn't a question of deciding not to. I mean, I got really sick…. I was in the middle of my addiction. I was homeless… and by the time I came out here and I actually wanted to get treated, I was undetectable. (Participant 132, Phoenix)
Still trying to get treatment	3 (8.8)	I was at the gastroenterology here in Yuma, and the lady was very helpful. I think it was more of the insurance that didn't want to help me. But so I did my six months clean, which is required, and I was good. I was able to apply for it, and I got denied for some clinical error or something. I had to apply in three months, or like appeal like in three months. And this kind of bugged me out, because they denied me because my liver wasn't damaged enough. So I imagine that there's a priority, right? That maybe some people that have it worse to get it treated. But in my letter, it said, “No, your liver is too damaged. We cannot help you with the treatment right now.” (Participant 116, Somerton) I'm actually trying to get on board so I can get the cure, but I haven't been able to get any information on that, to be honest.….and it just seems like people keep sending me or telling me, "Well, you're not eligible because of this or that." …. I feel like on a daily basis, I guess it affects me all the time. I'm tired and run down and my energy levels are low. (Participant 113, Phoenix) I had 60 days sober when I was at [MOUD clinic], and I had an appointment and I was just about to get my prescription for it and I ended up... they ended up drug testing me one more time and I ended up failing it because I ended up having an OxyContin in my system. That was the only experience I had and the closest I've ever came to getting the cure, and I'm hoping soon that I can get it. (Participant 41, Tucson)
Accessing treatment		
In treatment now	2 (5.9)	Well, yeah, it was difficult. Now I'm getting screened monthly, and my UAs have to pass, stay clean. So for them to do it... if I come out dirty, they won't start [the treatment]. So that's why I'm in the [sober living] houses over here. I have to be clean to stay there. They UA us randomly. (Participant 44, Yuma)
Cured/completed treatment	8 (23.5)	Participant: yeah. I got it done in prison, and it was very expensive. I took two pills a day.… I had no side effects. (Participant 22, phoenix) It was pretty easy. I found out I had it. I called my PCP. He referred me to a gastroenterologist... He did the testing and I went through all these different kinds of screenings to find out like, what type I had. How long. If it's caused any permanent damage. I was lucky I caught it early and I received treatment. I just had to do a couple of extra tests for the insurance. And then they sent me the medication through the mail and I took it every day at the same time each day for like eight weeks. (Participant 29, Tucson)

HCV, hepatitis C virus; MOUD, medication for opioid use disorder; PCP, primary care physician; UA, urine analysis.

Accessing curative treatment for HCV proved to be a challenge for some participants owing to sobriety requirements, complicated referrals, and insurance requirements. Repeated denials by insurance companies or HCV treatment providers appeared to impact the desire to continue seeking curative treatment according to those who were still trying to get treatment or had been unable to get it but were still hoping for it. In addition to the barriers listed earlier, there was reported fear of the treatment itself. Three people indicated that they had tried repeatedly to get curative treatment for HCV.

So I got screened, and then I got referred to another place, and then kept getting referrals. And then those appointments were always set out so far, and then the first that they would do obviously is check my levels and all that… And I got to see that my results were that my hep C was bad… like the levels were high enough so that I could get treatment, but my sobriety wasn't high enough, you know what I'm saying? So I got really discouraged at that point, and I was like, “Eff this. I don't care enough.” You know what I mean?....And I was actively using…. And then there was a time that I came back and it was maybe two months without any drugs or alcohol, and it wasn't good enough. I hadn't fulfilled those expectations to receive treatment. Hepatitis C is something that you can get cirrhosis, or everything can lead to cancer. And it's just like, oh, because I already have another disease of addiction where I can't stop... So yeah, and I got really discouraged and really bitter and hateful to the whole thing. (Participant 80, Prescott, AZ)

### Hepatitis C Virus Cascade

**Figure 1:**
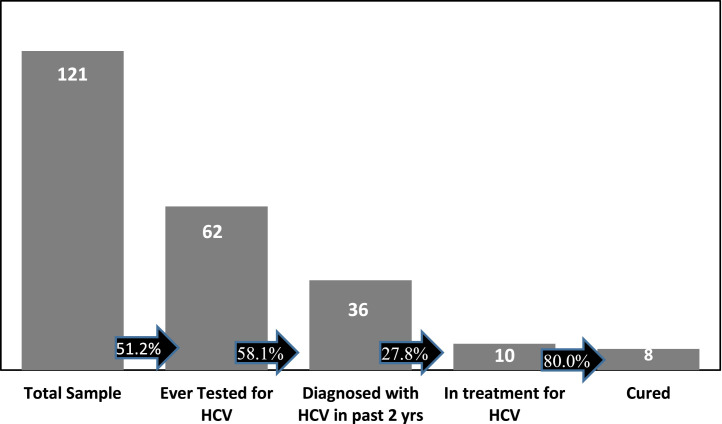
Hepatitis C cascade among people on methadone or buprenorphine during COVID-19, Arizona 2021. HCV, hepatitis C virus; yrs, years.

Eliminating HCV involves understanding the connection between screening, treatment, and cure. This allows public health interventions to be effectively targeted to those who need them most. Figure 1 displays how the interview cohort experiences of HCV testing and treatment arrayed along the adapted hepatitis C cascade.^24^

As shown in Figure 1, 54.8% of those ever testing for HCV reported being diagnosed in the past 2 years, but <30% of them were in treatment. Another 23.5% reported unsuccessful attempts to access curative treatment (Table 3).

## DISCUSSION

To our knowledge, this study was the first attempt to characterize an HCV testing and treatment experience and to array it along the HCV cascade in Arizona among people in OUD treatment and during the COVID-19 pandemic. Although the study cohort was small (*n*=121), there were a few key observations that may be useful to public health and policy partners as Arizona strives to eliminate HCV. As noted by Falade-Nwulia et al.,^6^ there is a need for more recent research on screening outcomes from MOUD providers, especially since the advent of recent curative treatments. Our study contributes in small part to this literature.

Identifying people who need curative treatment for HCV may be possible in OUD treatment settings as long as testing is offered consistently to all patients according to HCV screening guidelines. This means offering screening regularly and not just once during treatment, in recognition of polydrug use grounded in a harm-reductive approach. Such a harm-reduction perspective includes thinking that people can be in treatment for one substance and continue to use others because those drugs are not problematic for them. Thus, screening is not a one-and-done scenario unless a person seeks and obtains curative HCV treatment. So as with the extant literature, we have also shown that HCV screening by substance use disorder treatment providers identifies health needs among their patients.^25^

Measures of screening efforts could include the percentage of methadone and buprenorphine patients reporting a previous HCV test of >6 months without seeking curative treatment. It is apparent that OUD providers reach a portion of the population who need curative treatment, as evidenced by the high HCV positivity rate in this cohort. OUD provider-based screening, paired with community-based screening, may help to advance Arizona's understanding of who needs curative HCV treatment and that MOUD providers are in a unique position to help navigate patients to curative treatment. This moves beyond efforts to merely identify people who need curative treatment. Systems of navigation and patient advocacy must be developed to manage through the barriers with patients who have been traumatized by the healthcare system.

These facts notwithstanding, a central issue about whether OUD providers should offer screening and treatment involves the culture of treatment in OUD clinics and how it diverges from healthcare settings. As shown by Rance and Treloar,^18^ the culture of methadone clinics in New South Whales is one of minimal engagement and limited care in the midst of a carceral and surveillance-oriented framework of treatment. The same is true in the U.S. However, as they found, the integration of healthcare service provision for HCV testing and treatment by separate staff functioned to move (somewhat) the OUD treatment clinic culture toward more of a healthcare model. Additional studies would have to be conducted and especially in the U.S. A study from the United Kingdom showed the cost effectiveness of a nurse navigation model in treatment settings, which could inform the development of a navigation component.^26^

The finding that <30% of people with HCV accessed treatment trails the findings in Maier and colleagues’^24^ 2015 study documenting a 77% linkage to HCV care. However, in that study, Maier et al.^24^ found that only 17% of the participants were treated with HCV antivirals. If curative treatment were not provided on site at the OUD clinics, navigation to care and advocacy when faced with treatment access barriers would be required. This is particularly important given the history of maltreatment of people who use drugs by healthcare providers and lack of civil protections.^10^^,^^27^

Although reported barriers to curative treatment reflect studies elsewhere, Arizona has a state-specific task of addressing each barrier with structural and operational interventions. For example, in 2021, Arizona's Medicaid program lifted the sobriety barrier, but it remains unclear whether this policy barrier has been removed system wide in practice. Liver disease severity is a component of the current requirements, but it remains unclear whether specialists (required for treatment now) adhere to current or outdated standards.^28^

The complexity of accessing curative HCV treatment is put into sharp relief when considering that people who use drugs face terrible challenges to healthcare access generally.^29^^,^^30^ Furthermore, trust in one's OUD provider is not a given, owing to carceral constructions of treatment for substance use disorders, including patient surveillance and control of life patterns to govern access to treatment (especially with methadone).^15^^,^^16^ Investigating the feasibility and acceptability of HCV treatment navigation or even provision through OUD providers in Arizona would greatly inform avenues for elimination. This would be especially helpful if the state of Arizona would, in policy and practice, simplify treatment access and eliminate the need for specialist referrals and burdensome preauthorization and assure financial coverage beyond Medicaid for this public health priority. HCV treatment navigation by trusted harm-reduction partners would be necessary but not sufficient, given the broken and often discriminatory healthcare system that challenges the very goal of HCV elimination.

### Limitations

Data gathered for this study were self-reported by people who were MOUD patients during COVID-19 and are therefore based on recall. The study did not verify reported screening or treatment access using medical records. Furthermore, because the sample included 121 people, it may not represent the population of people on MOUD in Arizona during COVID-19.

## CONCLUSIONS

Structural barriers continue to prevent curative hepatitis C virus treatment access. However, there are opportunities to create pathways for screening and treatment navigation within methadone and buprenorphine treatment settings because these providers treat patients who are largely undiagnosed or untreated for hepatitis C virus. Future studies should design and test patient-empowered models of HCV testing and treatment in MOUD settings for provider and patient implementation acceptability and treatment outcome impact.
